# The Moral Side of Dentistry

**Published:** 1901-06-15

**Authors:** Geo. W. Clapp

**Affiliations:** Cygnet, Ohio


### THE MORAL SIDE OF DENTISTRY.

BY GEO. W. CLAPP, D.D.S., CYGNET, OHIO.

*Some Qualifications for Dentistry.*—Among the vocations of life, some, notably law, medicine and theology, have been set apart with the title of Learned Professions. Of late years dentistry has sought to take equal rank with these, basing its claims on the grounds of its present status and its probable future. The freest recognition of these claims is not dependent, as some have thought, upon the consent of the other professions; it is wholly dependent upon the members of the dental profession. When dentistry, as practiced by the average dentist, merits the respect usually accorded the learned professions, public opinion of dentistry as a trade will pass away, and it will be accorded the place it seeks. For the status of any profession is the average status of the men who are in it, and its future is the average future of its followers.

Separated from its practitioners and written down as a list of principles which might be applied and results which might be achieved, dentistry is not, so far as the practical issues of life are concerned, a profession; it is one in theory only. But when these principles are wrought into deeds, he who applies them takes rank according to his application. If his knowledge of the known principles be adequate; if his application be intelligent and skillful, and his service be marked by a spirit of loyalty to himself and his patient, the man and his calling take professional rank; otherwise they do not. Thus each practitioner determines whether dentistry is, or is not, a profession so far as he is concerned, and largely determines whether, in the eyes of those he serves, dentistry ranks among the learned professions, or is merely a trade. No great number of outsiders, certainly not people in general, are banded together to deprive dentistry of general recognition and respect as a learned profession. Dentistry is rated according to the average of its practitioners, and the fact that a few great leaders are carrying banners far in advance of the host, will not entitle the host to advanced recognition until it has moved up to its proper position.

The leaders of the profession have recognized this and have established colleges in an effort to secure for the average dentist adequate intellectual equipment. Doubtless they also grasped another idea which lies at the very threshold of their work, but which, from its inherent difficulty of application, they may have seen no better way of applying than the one now in use.

The idea is that there must be qualifications *for* dentistry before there can be qualification *in* dentistry; that the professional man is born, not made; that unless nature has qualified a man to begin with, no human preparation can make of him a truly professional man. So far as I know, no practical application of this principle of selection, other than entrance examinations in certain branches, is now made, even in our leading schools. In this and succeeding papers an effort will be made to determine what this principle of natural selection is which fits a few men for dentistry, (fewer by far than are entering) and to outline a plan by which our colleges may apply it in the selection of students.

The difference between the professional man and the non-professional is a difference of spirit. There may, and probably will, be a difference in education, but the spirit difference is, and will always be, chief. As they face the same problems, and perhaps serve the same people, they will do so from standpoints more effectually severed than the poles. There is a separation between them which the professional man will not cross, which the non-professional man can not; which instinctively bestows on one man high rank and place, and with equal effectiveness brands the other a tradesman. It is a difference of spirit, invisible, intangible, yet more actual and mighty than any other difference whatsoever.

It may well be asked, "What is this Professional Spirit on which so much responsibility is laid, which stands at the parting of the ways and divides men, inexorably, to the right hand and the left, without which no man can cross the barriers between a trade and a profession, and the possession of which prohibits a man turning his profession into a trade for the sake of gain?"

It is not easy to go behind the screen of life, as we see it in the acts of others, and unfold that spirit whose workings so sharply differentiate one man from another. It will be sufficient for our purposes if we can trace the workings of this Professional Spirit in those lives which it dominates, and can deduce the lessons necessary for its culture and exercise in our own lives.

As a young professional man I have in mind pictures of two men who are my ideal dentists. Both are old men; they have grown to years and honor in the profession. Both are striking men because of one fact; one can not look at them and not know that they are men of clean lives. A stranger might be justifiedin asking about their technical skill, but no one need ask about their characters ; after a look at their faces such questions would be superfluous. You would know instinctively that any woman or child was as safe in their offices as at home. I have heard many tributes paid to each man ; their professional children have risen up and called them blessed. Yet, so far as I remember, not one tribute has been paid because of surpassing professional knowledge. Hundreds of young men look up to them as I do, read their words with affectionate interest, and rejoice at the honors heaped upon them, not because they know more than all other men, but because each has added to adequate professional knowledge one thing more—the crown of manhood—a beautiful character.

The first and greatest outworking of the Professional Spirit is found in this quality of character. Probably this degree of it is possible only in the harvest time of life ; but this quality is possible to each young man, according to his capacity, if he will deliberately nurture the promptings of this spirit until it is master of his life.

Of such character it is not too much to say that it is fundamental, and absolutely essential to the truly professional man. To it might be applied, almost literally, the words of the Apostle to the Gentiles, "Other foundation can no man lay than is laid, which is character." "On this foundation men build gold, silver, precious stones, wood, hay, stubble." This, then, must the young man who would be truly professional, seek first ; and by this, first of all, his fellows have right to judge him. If he fail in this qualification no others will avail ; failure here should be sufficient to bar him from the profession.

Having satisfied the first great qualification, there remains another almost equally great. It is that the aspirant shall be of such cast and capacity of mind as shall fit him to accept the trust of a profession, and to become a leader of his fellow-men.

As to cast and capacity of mind. There seems to be a widespread notion that, given a youth of good character, average ability, and industrious habits, you can educate him to be anything he may choose. However true such a theory might be under ideal conditions, it does not work well in practice. The years of specialization are few and precious, and to him alone whom nature has qualified will they be productive of more than mechanical drill. Perhaps this notion is strengthened by a very common misunderstanding of what education means. Many think it means to fill an empty brain with facts, so that the young man may have them on hand, ready-made, as a merchant keeps shoes. Education means what the words from which it is derived mean, "to draw out," and relates to the drawing out and developing of the faculties of the mind. Obviously you can not draw out of the mind what is not there, and if a faculty is there in very limited degree its education will be slow and its final stature inconsiderable. This has been generally recognized by teachers. Fowler says: "As we are born, so, to a great extent, must we remain. Education may greatly strengthen or weaken organs already created, yet can create nothing." Ruskin writes: "Men have it determined for them, when they are born, what they shall be * * * currant out of apricot, great man out of small, never yet did art or effort make."

There is a cast of mind, sometimes spoken of as the "scientific cast," which naturally qualifies a man for a profession. It is marked by a considerable development of the perceptive faculties; by the ability to perceive conditions which, though not visible to the eye, may be clearly seen by the trained mind, and by the power to reason from the things perceived to intelligent and reasonable conclusions. In some men these powers are habitually exercised; in some they lie dormant; in some they are incapable of development. These last see the thing set before them, some more of it, some less, but by no effort of mind can they get behind the visible thing and grasp the point they need to know. It is evident that such men can hope for but little in dentistry.

Another characteristic of the scientific cast of mind is that it is pre-eminently the mind of the student. Its possessor is one who, by long hours of hard work, by years of pursuit, and by keen perception and close reasoning, advances little by little the boundaries of professional knowledge. It is he of whom the poet wrote:

"The heights by great men reached and kept,
Were not attained by sudden flight;
But they, while their companions slept,
Were toiling upward in the night."

To this cast of mind must be added capacity of mind, for capacity is not less important than direction. As a writer in the *Outlook* has well said, "The greatest difference between men is found in their varying degrees of capacity for growth. There are a great many young men who never fulfill the pledges of their youth because they lack this capacity for growth." It needs but a thought to tell the experienced dentist that unless the aspirant has this capacity for growth, unless he can perceive new things each day, and each day break down some old conclusion for a better one, he is not fitted for dentistry. It needs but the mention of how opinions are changing under the influence of such men as Black, Miller, Williams and others to show that the man whose nature it is to get into a rut and stay there is not born to a profession.

Finally, the aspirant must be of such a spirit as fits him to accept the trust of a profession and to be a safe leader of his fellow-man. Some one has said that a diploma is a pledge to learn for all, to work for all and to teach for all. To the mind of the born professional man his profession is a sacred trust, and he, as its missionary, takes rank with those, who, from age to age, go forth to proclaim the truth in all its varied forms. Certainly to be placed in advance of our fellows as their leaders, to be given charge of their health and lives, and to be admitted to the close relationships with their families which any profession sometimes requires, can not be considered by any right-minded man as other than a trust to be jealously guarded and to be deserved anew from day to day. Our relations with patients, especially in offices where no assistants are employed, are often very close. Sometimes for hours each day, and day after day we work for ladies, often in approximations which tend to break down many of the safeguards of ordinary association. They know that we can often detect by a glance things which are hidden from people in general, and the freedom thus engendered tends to break down more barriers. However much a man may know of dentistry, if he is, under such conditions, a menace to the happiness of any home, he is a moral leper, unfit to be distinguished and endorsed by the diploma of any reputable school. His moral laxity will so degrade the profession to all who see his depravity that no skill or knowledge on his part, or perhaps on the part of others, may ever be able to restore it to its proper place. But if he so elevate his profession to himself that his life and manner proclaim his thoughts to be on his work, and that alone ; if they proclaim an utter absence of impure thoughts and desires, he will elevate his profession and himself in the eyes of his patrons to a degree not otherwise possible.

It is safe to say that no man can elevate a profession to himselt or his patrons who views it merely as a means for making money ; and the young man who elects to practice dentistry because it will permit him to wear good clothes and earn a good living, is not a safe man to entrust with the honor of the profession. There is no better index of a man's spirit in this regard than his attitude toward his patient and his opportunity. If he view his patient merely as one whose needs require that he spend money for relief; if he view the patient's needs merely as an opportunity for securing so much money, and if, regardless of fitness, he prescribe a certain kind of work because he can make more money out of that than out of any other kind, he is entirely lacking in what I have sought to describe as the Professional Spirit. Such a man can not long maintain professional standing, even in his own eyes, and because of him the whole profession will receive no small set back.

If, on the other hand, he view his patient as one in need of skillful service ; if he view his opportunity as one for the exercise and advancement of the profession he loves ; and if he advise in this spirit he is one whose picture I have tried to sketch, a man qualified for the profession.

The characteristics, then, cast and capacity of mind, and a worthy conception of dentistry as a profession and not a trade, built upon a good character, are the qualifications with which nature endows the man whom she fits for dentistry. On this foundation must be built adequate general and special training. But no amount of training *in* dentistry will supply the lack of qualifications *for* dentistry.

